# Danshen-Shanzha formula for the treatment of atherosclerosis: ethnopharmacological relevance, preparation methods, chemical constituents, pharmacokinetic properties, and pharmacological effects

**DOI:** 10.3389/fphar.2024.1380977

**Published:** 2024-06-07

**Authors:** Qiong Xu, Zhe Yu, Meng Zhang, Tian Feng, Fan Song, Haifeng Tang, Siwang Wang, Hua Li

**Affiliations:** ^1^ Department of Chinese Materia Medica and Natural Medicines, School of Pharmacy, Air Force Medical University, Xi’an, China; ^2^ Department of Pharmaceutical Analysis, School of Pharmacy, Air Force Medical University, Xi’an, China; ^3^ School of Graduate Studies, Air Force Medical University, Xi’an, China

**Keywords:** danshen-shanzha formula, herb pair, *radix salvia miltiorrhiza*, *fructus crataegi*, atherosclerosis

## Abstract

Danshen-Shanzha Formula (DSF) is a well-known herbal combination comprising *Radix Salvia Miltiorrhiza* (known as Danshen in Chinese) and *Fructus Crataegi* (known as Shanzha in Chinese), It has been documented to exhibit considerable benefits for promoting blood circulation and removing blood stasis, and was used extensively in the treatment of atherosclerotic cardiac and cerebral vascular diseases over decades. Despite several breakthroughs achieved in the basic research and clinical applications of DSF over the past decades, there is a lack of comprehensive reviews summarizing its features and research, which hinders further exploration and exploitation of this promising formula. This review aims to provide a comprehensive interpretation of DSF in terms of its ethnopharmacological relevance, preparation methods, chemical constituents, pharmacokinetic properties and pharmacological effects. The related information on Danshen, Shanzha, and DSF was obtained from internationally recognized online scientific databases, including Web of Science, PubMed, Google Scholar, China National Knowledge Infrastructure, Baidu Scholar, ScienceDirect, ACS Publications, Online Library, Wan Fang Database as well as Flora of China. Data were also gathered from documentations, printed works and classics, such as the Chinese Pharmacopoeia, Chinese herbal classics, *etc.* Three essential avenues for future studies were put forward as follows: a) Develop and unify the standard preparation method of DSF as to achieve optimized pharmacological properties. b) Elucidate the functional mechanisms as well as the rationality and rule for the compatibility art of DSF by focusing on the clinic syndromes together with the subsequent development of preclinic study system *in vitro* and *in vivo* with consistent pathological features, pharmacokinetical behaviour and biomarkers. c) Perform more extensive clinical studies towards the advancement of mechanism-based on evidence-based medicine on the safety application of DSF. This review will provide substantial data support and broader perspective for further research on the renowned formula.

## Graphical Abstract

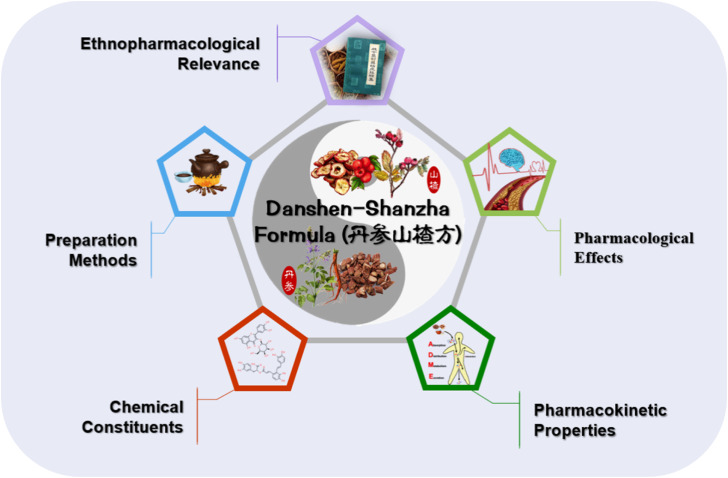

## 1 Introduction

Ancient Chinese developed the expertise of employing natural materials to alleviate disease as a result of their lengthy battle for surviving against a variety of adverse conditions (Muszynska et al., 2015). There are numerous records about the use of natural plants, animals, and minerals to treat diseases in the classics of traditional Chinese medicine, and these materials are collectively referred to as Traditional Chinese Medicine (TCM) (Ai et al., 2023). Traditional Chinese Medicine has constructed the foundations of a sophisticated traditional medical system through thousands of years of medication discovery, experience accumulation as well as knowledge preservation, and served as a safeguard for the health of the Chinese since ancient times (Lv, R. et al., 2022; Zhang et al., 2023). In the past few decades, as a result of the increased attention given to TCM around the world, it has become an increasingly essential healthcare system not just in China but also globally, which may stably provide innovative therapies for patients with life-threatening diseases in the modern society (Xing and Liu, 2021).

Early in its history of medical practice, traditional Chinese medicine often relied on single herb to treat diseases (Oravecz and Meszaros, 2012). However, with the steady collection of therapeutic experience, TCM practitioners have progressively become aware of the complexities of pathogenesis for the diseases, and a variety of creative compatibility art for herbs have been proposed in an effort to improve therapeutic benefit while minimizing adverse effects (Ji et al., 2018). In the earliest Chinese pharmacy book known as “Shen Nong Ben Cao Jing,” the theory of “seven features of compatibility” identifies single application, potentiation, assistance, toxicity restraint, detoxification, inhibition, and incompatibility as the seven types of interactions that can occur between herbs (Yang et al., 2018). As a result, multi-herb therapies became an integral part of traditional Chinese medical systems and since then been practiced for decades of centuries throughout China and other nations. Multi-herb formulation invokes the concept of system-based strategies that integrate multiple inputs to address the complexities of a disease condition (Li et al., 2016; Tang et al., 2022). As the core of the culture for traditional Chinese medicine, more than one hundred thousand formulations have been amassed over the past 2000 years (Li and Zhang, 2008). It is noteworthy that a special group, called herb pairs, served as a crucial part in the development of the theory of multi-herb formulation. Herb pairs is a delicate combination consisting of two relatively fixed herbs throughout clinical use, which can be regarded as the most basic and simple forms of multi-herb formulation (Wang, S. et al., 2012). In the Treatise on Exogenous Febrile and Miscellaneous Diseases (210 AD), one of the most valuable classics of herbal formulas, one hundred and forty-seven herb pairs have been documented, and over forty formulas were consisted based on two herbs. To this day, a great deal of preparations derived from herb pairs are routinely documented in the latest version of Chinese pharmacopoeia (2020 edition). Among the compatibility art of herb pairs, potentiation and assistance are the most common compatibilities. Synergistic effects of herb pairs are able to be accomplished through utilizing a pair of herbs with particular components of unique pharmacodynamic characteristics or pharmacokinetic properties. As an instance, one active component may improve the therapeutic effect of another active component through activating specific pathways, or by modulating its absorption, distribution, metabolism and excretion (ADME) (Zhang and Ren, 2017). These compatibilities have long been adopted in many noted herb pairs, such as Shuang Dan Fang (*Radix Salviae Miltiorrhizae* and *Cortex Moutan*) and Yuan Hu Zhi Tong Fang (*Rhizoma Coridalis* and *Angelica dahurica*) (Li et al., 2016; Liu et al., 2020).

Danshen-Shanzha Formula (DSF), derived from Danshen and Shanzha herb pair (DSHP) which composed of *Radix Salvia miltiorrhiza* (Chinese name: Danshen) and *fructus crataegi* (Chinese name: Shanzha), is one of the most classic herb formula for mutual potentiation and has been extensively applied for the therapy of blood stasis conditions with long-term clinical experience. Pharmacological studies have demonstrated that DSF could reduce lipid level, free radical scavenging, reduce endothelial dysfunction, inhibit inflammation (Zhang et al., 2019). In modern medicine, the anti-hypolipidemic effect of DSF has been clearly demonstrated, and multiple dosage forms of patent medicine derived from this formula in clinical applications, such as oral particles, decoctions and powder, are all primarily indicated for atherosclerotic conditions (Liu et al., 2012; Gu et al., 2016; Anonymous, 2019). Despite several breakthroughs achieved in the basic research and clinical applications of DSF over the past two decades, there is a lack of comprehensive reviews summarizing its features and research, which hinders further exploration and exploitation of this promising formula.

Herein, with the aim of providing advantageous details for the scientific studies and modern applications of DSF, the research status of DSF were summarized in terms of its ethnopharmacological relevance, preparation methods, chemical constituents, pharmacokinetic properties and pharmacological effects by searching related keywords through China National Knowledge Infrastructure (CNKI), PubMed, Web of Science, along with other databases. It is expected to enlighten the comprehensive and deeper knowledge of DSF, thus providing innovative idea for system studies of this herbal combination in the future.

## 2 Ethnopharmacological relevance

### 2.1 History of Danshen-Shanzha Formula (DSF)

Danshen-Shanzha formula (DSF), a form of prescription for danshen (the root of *Salvia miltiorrhiza* Bge., DS) and shanzha (the fruit of *Crataegus pinnatifida* Bge., SZ) herb pair, was originally used in folk medicine since the Han Dynasty in the form of empirical formula. It was used clinically by Dr. Jin-MO Shi in the late Qing Dynasty, who believed that this combination had the effect of promoting Qi and blood circulation, removing blood stasis and relieving pain. This traditional Chinese herbal prescription was officially recorded in the book “Shi Jin-Mo’s Clinical Experience on Medicine” in 1982 (Lv, 2005), written by Dr. Jing-Shan Lv, the student of Dr. Shi. Subsequently, this formula was developed into a modern preparation “Xiaoyu Jiangzhi Capsule” (SFDA approval number Z20060427), composed of total phenolic acid extract of salvia miltiorrhiza and total triterpene acid extract of hawthorn, which is primarily utilized as a preventative medicine for hyperlipidemia (Wu et al., 2022). Furthermore, DSF has also been widely served as the fundamental herb pair in numerous traditional complex formulas and patent medicines for clinical treatment of cardiac and cerebral vascular diseases, primarily aiming at promoting blood circulation and removing blood stasis. Examples include Xin Ke Shu (Yang et al., 2018), Xin Shu Bao (Zhang et al., 2022) and Shuan Tong Ling (Mei et al., 2017).

### 2.2 Traditional background of the constituent herbs of DSF

Danshen is the dried radix and rhizome of *Salvia miltiorrhiza Bge*. The usual harvest time is in the stage from early October-November or late spring before germination (He et al., 2010b), which has been considered to be the ideal period for the accumulation of the active components, including salvianolic acid, tanshinone, and aliphatic acid (Zeng et al., 2017). The sources of both wild and cultivated DS have a broad distribution over Shandong, Henan, Shaanxi, and Sichuan province of China, among which Shandong enjoys a good reputation both at mainland and abroad, with advantage on per unit output, drug potency, root color and appearance (He et al., 2010a). According to Traditional Chinese Classics, DS is documented as the herb that have the beneficial properties of promoting blood circulation, removing blood stasis, relieving pain through the meridian, clearing the heat and removing irritations, and cooling blood and eliminating blemishes (Liu et al., 2020). From Han Dynasty (202 BC) to modern society, DS has been extensively utilized in conjunction with various herbs to address a wide range of health issues, including diabetes, hepatocirrhosis, osteoporosis, and particularly cerebrovascular as well as cardiovascular diseases (Zhang and Ren, 2017). Studies on phytochemistry have revealed that DS contains a variety amount of phenylpropionic acids and diterpenequinones (Liu et al., 2023).

Shanzha, commonly considered to be the fruit of *Crateagus pinnatifida* Bge. and its variant “Shan-Li-Hong” (*Crateagus pinnatifida* Bge. var. major N. E. Br.), owing to its enormous fruit, distinctive tasty, and characteristic sourness. Moreover, the above mentioned two source of Shanzha are catalogued in the latest version (2020 edition) of Chinese Pharmacopoeia, as a kind of TCM for treating cardiovascular and gastrointestinal disorders (Li et al., 2023). SZ is widespread in northern China, the best harvest time is in the fructescence stage in autumn. SZ has a long tradition for the medical application throughout the Chinese history. According to the Compendium of Materia Medica (Chinese name: Ben Cao Gang Mu), a renowed TCM masterpiece, the initial recorded clinic application of SZ has been recorded as the therapy for the symptom of “disorder of Qi and blood,” such as dyspepsia, cardiodynia and *postpartum* blood stasis, ets., dating back to 1552 AD. Over the past several decades, studies have revealed that SZ exerts a variety of pharmacological beneficial impacts on circulatory, digestive and endocrine systems. Moreover, to date, around 150 chemicals have been isolated and identified from SZ, including phenylpropanoids, triterpenic acids, flavonoids and polysaccharides (Ye et al., 2023). In addition to its traditional usage as a peptic agent, SZ has also been widely employed as foodstuffs in China and the Europe, including soft drink products, jams, juices, tinned goods, ingredients for wines, and a variety of sweet dishes. In recent years, SZ are receiving more and more interest in foodstuffs industry and medical field owing to its well acknowledged health advantages, such as the efficacy in decreasing serum cholesterol levels and lowering the risk of coronary heart disease (Pei et al., 2022).

### 2.3 Theoretical basis and clinical applications of DSF

In ancient texts of traditional Chinese medicine, atherosclerosis was not specifically labeled. Some medical practitioners categorized it under concepts such as “blood stasis” or “phlegm turbidity” based on its underlying causes, while others denoted it as “vessel obstruction” depending on the affected region (Yi et al., 2023). However, according to the clinical theory of traditional Chinese medicine, there is a prevailing consensus that the fundamental pathogenesis of atherosclerosis lies in Qi stagnation and blood stasis, as well as phlegm turbidity and food stagnation. This implies that the human vessels are vulnerable to the influence of pathogenic toxins, subsequently resulting in impaired circulation due to the occurrence of stagnation. Concurrently, the presence of blood stasis obstructs the veins, thereby exacerbating the hindrance of both Qi and blood flow, ultimately culminating in the progression of atherosclerosis (Li et al., 2024). Hence, traditional Chinese medical practitioners have endeavored to enhance the flow of blood in order to eliminate pathogenic toxins within the vessels, a practice referred to as “promote blood circulation and remove blood stasis.” This principle has emerged as a crucial strategy for treating atherosclerosis within the system of TCM (Li et al., 2022). Interestingly, DS has been found to enhance hemodynamic circulation and promote tissue regeneration, whereas ancient texts suggest that the effectiveness of DS is comparable to Si Wu Tang, indicating its potential for the blood-activating and stasis-resolving properties equivalent to the combination of *Radix Angelicae Sinensis*, *Radix Paeoniae Alba*, *Rhizoma Ligustici Wallichii*, and *Rhizoma Rehmanniae* (Yang and Ma, 2024). Therefore, in clinical applications of Chinese medicine, DS is widely utilized as a crucial ingredient in most of the classical prescriptions and patent medicines that primarily aim to promote blood circulation and resolve blood stasis, such as Danshen Decoction, Compound Danshen Dripping Pills, and Danhong Injection. On the other hand, in accordance with the principles of TCM, phlegm turbidity arises from spleen and stomach weakness. When the functions of these organs in the middle burner become abnormal, they are unable to effectively transport and disperse water and aliment, leading to excessive lipid accumulation in blood vessels and subsequent formation of turbid phlegm. Ultimately, this can impair blood vessel function and contribute to the development of atherosclerosis. Based on records from traditional Chinese medical classics as well as clinical experience, SZ not only possesses the capability of prompting blood circulation and inhibiting thrombus formation but also excels in enhancing gastrointestinal motility and reducing blood lipids. Consequently, in order to achieve the therapeutic effect of alleviating atherosclerosis, Dr. Shi has integrated the principles of promoting blood circulation and removing blood stasis with strengthening stomach function and eliminating turbidity through years of medical practice. The combination of DZ and SZ (known as DSF) was utilized, wherein SZ synergistically enhanced the efficacy of DS in promoting blood circulation and removing blood stasis. Additionally, SZ emphasized its potential in strengthening gastric function and reducing lipid levels for treating hyperlipidemia. The combination undeniably offers clear therapeutic benefits in the treatment of atherosclerosis associated with phlegm toxin and blood stasis syndrome (Lv, 2005; Li et al., 2023). Currently, DSF has been employed in the treatment of various manifestations of atherosclerosis in modern TCM, encompassing impaired endothelial function, disrupted lipid homeostasis, and unregulated immune-inflammatory reactions (Zhang, 2013; Zhang et al., 2016; Zhang et al., 2019; Liu et al., 2022). Notably, it has shown promising results in reducing the risk factors associated with atherosclerosis, such as high cholesterol levels and hypertension. Research have indicated that DSF can effectively lower LDL cholesterol while increasing HDL cholesterol levels, thereby improving overall lipid profile and reducing the likelihood of plaque formation in arteries (Gao et al., 2007; Zhang, 2009). Importantly, studies evaluating the safety characteristics of DSF revealing minimal occurrence of adverse reactions or side effects. This characteristic renders it an appealing option for long-term utilization without compromising patient health or causing undue harms (Bi et al., 2015; Wu, 2015; Gu et al., 2016; Zhang et al., 2022). Pharmacological research has indicated that DSF possesses the ability to effectively dilate blood vessels, promote blood circulation and improve cardiac function, thereby exhibiting promising therapeutic potential in the treatment of cardiovascular and cerebrovascular diseases, including angina pectoris, myocardial ischemic damage, and cerebral infarction (Wu, 2015; Xu et al., 2019; Wen et al., 2021). Additionally, extensive research has been conducted on formulas or preparations containing DSF, which have demonstrated numerous therapeutic benefits in the treatment of various health conditions. For example, Xin-Ke-Shu tablets have exhibited efficacy in managing arrhythmia (Yang, 2008), while Danshen-Shanzha Decoction has proven effective in treating non-alcoholic steatohepatitis (Wang and Guo, 2018). Tong-Mai-Hua-Zhuo Decoction has shown promise in controlling hypertension (Nan et al., 2017), and Jiang-Zhi-Qing-Nao Decoction has been explored for its potential benefits in managing hyperlipemia (Luan and Hao, 2012). Moreover, research suggests that DSF-containing formulas like Yi-Qi-Huo-Xue-Tong-Luo Decoction may play a role in improving outcomes for individuals with cerebral ischemic stroke (Qu, 2015). Furthermore, Shanzha-Danshen Decoction has been investigated for its potential therapeutic effects on coronary heart disease (Liu, 2010; Zhang et al., 2013b). The therapeutic benefits attributed to these aforementioned formulas or preparations are undeniably linked to the role of DSF in promoting blood circulation and removing blood stasis along with eliminating turbidity. Moreover, these therapeutic advantages highlight the adaptability and potential of formulations containing DSF as a complementary approach alongside conventional treatments. Nevertheless, additional research is still necessary for elucidating their mechanisms of action fully and optimizing their application in clinical practice.

## 3 Preparation methods

The majority of herbs in Chinese medicine are typically administered through water-based decoctions. For the original prescription written in the book Shi Jin Mo Dui Yao, the dose utilized is stated as 10–30 g DS+10–30 g SZ for a set of daily use, however the amount of water used in boiling of DSF did not spell out specifically. According to the ancient literature and usual decoction method of Chinese medicine, we considered a set of DS-SZ herbal mixture, should at least put in two bowls (approximately 600 mL) of water and decocted until the final volume is halved. The compatibility ratio of DS and SZ is normally adjusted by the physician based on the clinical condition, and the ratio of 1:1 has been used widely in the clinic.

On the other hand, improved preparation methods are routinely taken by investigators in preclinical research and have a standardized process. Among which, the preparation process developed by Zhang et al. (2013a) has been widely employed and referenced in several studies. According to the method, 1-kg DS and 1-kg SZ should be sliced followed by extracting and refluxing with eight times amount of 70% ethanol (*w*/*v*) for a duration of 2 h. Subsequently, resulting mixtures were subjected to two rounds of concentration using a rotary evaporator at a temperature of 40°C, resulting in the formation of a brown gum. This gum was then suspended in distilled water and subjected to chloroform extraction, which was repeated three times to yield both a chloroform extract and a water layer. The water layer was then subjected to centrifugation at 4°C with 3,500 r/min for 20 min, and the resulting supernatant was purified using a D101 macroporous resin column. Elution was carried out using varying concentrations of ethanol in a sequential manner, and the elutions were combined and concentrated under vacuum. Finally, the concentrated solution was freeze-dried to obtain the DSF extract lyophilized powder (200 g). Seven previous reported bioactive ingredients, including chlorogenic acid, procyanidin B2, (−)-epicatechin, rosmarinic acid, lithospermic acid, salvianolic acid B, and salvianolic acid A, were taken as the QC compounds to ensure the quality of the DSF lyophilized powder. However, danshensu, one of the most important hydrosoluble components of DS, was not detected in the DSF extract prepared by this method. Therefore, we believe that this preparation method still needs be improved in the future.

DSF could also been prepared by decocting the individual herbs separately. According to Bao et al. (2013) method, SZ was extracted twice with ten times the amount of 75% ethanol (w/v) in reflux for 1 h followed by concentrating to 1.0 g/mL (equivalent to crude drug). The concentrated extract will be purified by an AB-8 macroporous resin column and then rinsed with 70% ethanol. The elutions were taken apart, combined and concentrated in a vacuum chamber before being freeze-dried to obtain SZ lyophilized powder. DS lyophilized powder would be obtained by the same method. And these two kinds of lyophilized powder were mixed in the ratio of 4:5 to obtain DSF lyophilized powder. This kind of preparation methods has the benefit of preserving the chemical composition of the two herbs to a larger extent, but does not take into account for the potential chemical interactions that may occur when the two herbs are decocted together, and is somewhat different from the clinical preparation method recorded in ancient books.

## 4 Chemical constituents

It is generally known that the bioactivities of herbal remedies largely depend on the chemical constituents of the herbs. Thus, it is critical to accurately comprehend the material foundation of DSF with the aim to elucidate, inherit and develop the compatibility art of the herb pair. To obtain a thorough understanding for the phytochemical details of DSF, a brief introduction of the chemical constituents of Danshen and Shanzha will be provided respectively, after which comes the research progress on compatibility behavior for the constituents of their combination form DSF.

### 4.1 Chemical constituents in Danshen

**FIGURE 1:**
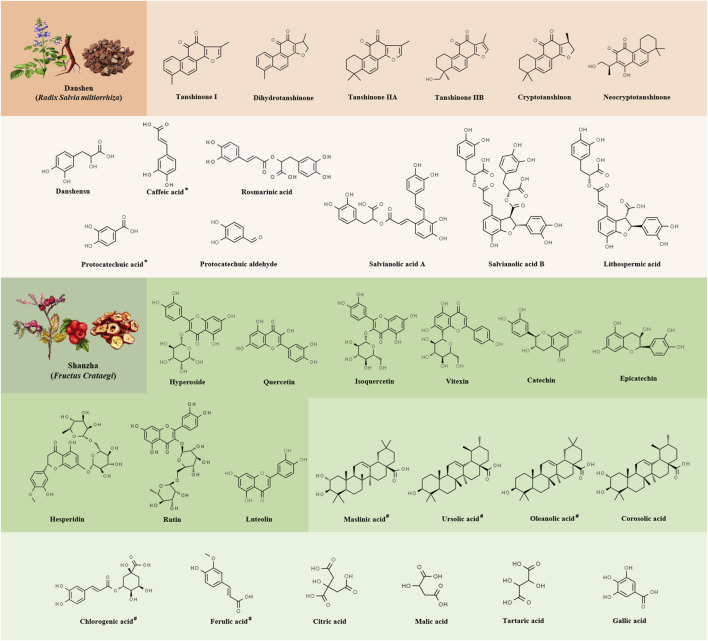
The chemical structures of the primary constituents exhibiting vascular activity in Danshen-Shanzha Formula (DSF). The different shades of orange from top to bottom represent the phenylpropionic acids (orange) and tanshinones (light orange) in Danshen. The different shades of green from top to bottom represent the flavonoids (green), tritieric acid (pale green) and phenylpropionic and organic acids (light green) in Shanzha. The asterisk (*) denotes the presence of the constituent in Shanzha as well, the number sign (#) denotes the presence of the constituent in Danshen as well.

Danshen (DS, the root of *Salvia miltiorrhiza Bge*.) was proven to have many active ingredients, including diterpenoids, triterpenoids, lactone, nitrogenous, phenolic acids, and other compounds (Jia et al., 2019). According to the Chemistry Database, over than a hundred compounds have been identified from DS until so far, including water-soluble phenylpropionic acids such as salvianolic acid A/B/C/D/E/F/G, lipid-soluble phenanthraquinones such as tanshinone I/IIA/IIB/V/VI, dihydrotanshinone І, tanshindiol A, miltirone, dehydromiltirone and isotanshinone, etc (Su et al., 2015; Yuan et al., 2020). Readers who are interested in obtaining further information regarding the chemical structures of DS compounds are advised to refer to the specialized review of *Salvia miltiorrhiza Bge* conducted by Wang et al. (Wang et al., 2017). Within the scope of this current review, we have exclusively presented a compilation of the primary compounds related to ingredients of DS (Table 1), along with the principal chemical structures that have been reported in literature concerning anti-atherosclerotic activity (Figure 1).

**TABLE 1:** Main chemical constituents and relative detection methods of Danshen-Shanzha Formula (DSF).

No.	Constituents	Extraction solvent	Detection method	Source	References
Tanshinones					
1	Tanshinone I	70% methanol	HPLC-PAD/HPLC-MS	Danshen	Liang et al. (2017)
2	Dihydrotanshinone	50% ethanol	HPLC-MS/MS	Danshen	Pang et al. (2018)
3	Tanshinone IIA	supercritical CO_2_ fluid	HPLC-MS	Danshen	Yuan et al. (2020)
		70% methanol	HPLC-PDA/HPLC-MS	Danshen	Liang et al. (2017)
		50% ethanol	HPLC-MS/MS	Danshen	Pang et al. (2018)
4	Tanshinone IIB	70% methanol	HPLC-PDA/HPLC-MS	Danshen	Liang et al. (2017)
		50% ethanol	HPLC-MS/MS	Danshen	Pang et al. (2018)
5	Cryptotanshinone	supercritical CO_2_ fluid	HPLC-MS	Danshen	Yuan et al. (2020)
		70% methanol	HPLC-PDA/HPLC-MS	Danshen	Liang et al. (2017)
		50% ethanol	HPLC-MS/MS	Danshen	Pang et al. (2018)
6	Neocryptotanshinone	50% ethanol	HPLC-MS/MS	Danshen	Pang et al. (2018)
7	Methyl tanshinonate	70% methanol	HPLC-PDA/HPLC-MS	Danshen	Liang et al. (2017)
		50% ethanol	HPLC-MS/MS	Danshen	Pang et al. (2018)
8	Tanshindiol B	70% methanol	HPLC-PDA/HPLC-MS	Danshen	Liang et al. (2017)
9	Tanshindiol C	70% methanol	HPLC-PDA/HPLC-MS	Danshen	Liang et al. (2017)
10	3*a*-Hydroxymethylenetanshinquinone	70% methanol	HPLC-PDA/HPLC-MS	Danshen	Liang et al. (2017)
11	Tanshinol B	50% ethanol	HPLC-MS/MS	Danshen	Pang et al. (2018)
		70% methanol	HPLC-PDA/HPLC-MS	Danshen	Liang et al. (2017)
12	Tanshinaldehyde	70% methanol	HPLC-PDA/HPLC-MS	Danshen	Liang et al. (2017)
13	Trijuganone B	70% methanol	HPLC-PDA/HPLC-MS	Danshen	Liang et al. (2017)
14	Danshenxinkun A	70% methanol	HPLC-PDA/HPLC-MS	Danshen	Liang et al. (2017)
15	Miltirone	70% methanol	HPLC-PDA/HPLC-MS	Danshen	Liang et al. (2017)
		50% ethanol	HPLC-MS/MS	Danshen	Pang et al. (2018)
16	Neoprzewaquinone A	50% ethanol	HPLC-MS/MS	Danshen	Pang et al. (2018)
17	Danshenxinkun B	50% ethanol	HPLC-MS/MS	Danshen	Pang et al. (2018)
18	Hydroxytanshinone IIA	50% ethanol	HPLC-MS/MS	Danshen	Pang et al. (2018)
19	Methyl dihydronortanshinonate	70% methanol	HPLC-PDA/HPLC-MS	Danshen	Liang et al. (2017)
		50% ethanol	HPLC-MS/MS	Danshen	Pang et al. (2018)
20	Przewaquinone B	50% ethanol	HPLC-MS/MS	Danshen	Pang et al. (2018)
21	Hydroxycryptotanshinone	50% ethanol	HPLC-MS/MS	Danshen	Pang et al. (2018)
22	Salvianonol	50% ethanol	HPLC-MS/MS	Danshen	Pang et al. (2018)
23	Dehydromiltirone	50% ethanol	HPLC-MS/MS	Danshen	Pang et al. (2018)
24	Dehydrotanshinone IIA	50% ethanol	HPLC-MS/MS	Danshen	Pang et al. (2018)
Phenylpropanoids					
25	Danshensu	50% ethanol	HPLC-MS/MS	Danshen	Pang et al. (2018)
		supercritical CO_2_ fluid	HPLC-MS	Danshen	Yuan et al. (2020)
		70% methanol	HPLC-PDA/HPLC-MS	Danshen	Liang et al. (2017)
26	Protocatechuic acid	supercritical CO_2_ fluid	HPLC	Danshen	Yuan et al. (2020)
		70% methanol	HPLC-PDA/HPLC-MS	Danshen	Liang et al. (2017)
		50% ethanol	HPLC-MS/MS	Danshen	Pang et al. (2018)
		80% methanol	HPLC-PDA	Shanzha	Sun et al. (2021)
27	Protocatechuic aldehyde	supercritical CO_2_ fluid	HPLC-MS	Danshen	Yuan et al. (2020)
		70% methanol	HPLC-PDA/HPLC-MS	Danshen	Liang et al. (2017)
		50% ethanol	HPLC-MS/MS	Danshen	Pang et al. (2018)
28	Caffeic acid	70% methanol	HPLC-PDA/HPLC-MS	Danshen	Liang et al. (2017)
		50% ethanol	HPLC-MS/MS	Danshen	Pang et al. (2018)
		80% methanol	HPLC-PDA	Shanzha	Sun et al. (2021)
29	Rosmarinic acid	50% ethanol	HPLC-MS/MS	Danshen	Pang et al. (2018)
30	Salvianolic acid A	70% methanol	HPLC-PDA/HPLC-MS	Danshen	Liang et al. (2017)
		water	HPLC	Danshen	Chen et al. (2013)
		50% ethanol	HPLC-MS/MS	Danshen	Pang et al. (2018)
31	Salvianolic acid B	supercritical CO_2_ fluid	HPLC-MS	Danshen	Yuan et al. (2020)
		70% methanol	HPLC-PDA/HPLC-MS	Danshen	Liang et al. (2017)
		water	HPLC-PDA	Danshen	Chen et al. (2013)
		50% ethanol	HPLC-MS/MS	Danshen	Pang et al. (2018)
32	Salvianolic acid C	50% ethanol	HPLC-MS/MS	Danshen	Pang et al. (2018)
33	Salvianolic acid D	water	HPLC/Q-TOF-MS	Danshen	Wu et al. (2018)
34	Salvianolic acid E	70% methanol	HPLC-PDA/HPLC-MS	Danshen	Liang et al. (2017)
		50% ethanol	HPLC-MS/MS	Danshen	Pang et al. (2018)
35	Salvianolic acid F	70% methanol	HPLC-PDA/HPLC-MS	Danshen	Liang et al. (2017)
		50% ethanol	HPLC-MS/MS	Danshen	Pang et al. (2018)
36	Salvianolic acid G	70% methanol	HPLC-PDA/HPLC-MS	Danshen	Liang et al. (2017)
37	Salvianolic acid H	50% ethanol	HPLC-MS/MS	Danshen	Pang et al. (2018)
38	Salvianolic acid I	50% ethanol	HPLC-MS/MS	Danshen	Pang et al. (2018)
39	Salvianolic acid L	70% methanol	HPLC-PDA/HPLC-MS	Danshen	Liang et al. (2017)
40	ProcyanidinB2	water	HPLC-PDA	Danshen	Chen et al. (2013)
41	Isosalvianolic acid C	50% ethanol	HPLC-MS/MS	Danshen	Pang et al. (2018)
42	Chlorogenic acid	water	HPLC-PDA	Danshen	Chen et al. (2013)
		75% ethanol	UPLC/ESI-TOF-MS	Shanzha	Qiao et al. (2014)
43	Ferulic acid	50% ethanol	HPLC-MS/MS	Danshen	Pang et al. (2018)
		80% methanol	HPLC-PDA	Shanzha	Sun et al. (2021)
44	Isoferulic acid	50% ethanol	HPLC-MS/MS	Danshen	Pang et al. (2018)
45	Lithospermic acid	water	HPLC-PDA	Danshen	Chen et al. (2013)
		70% methanol	HPLC-PDA/HPLC-MS	Danshen	Liang et al. (2017)
46	Dimethyl lithospermate	50% ethanol	HPLC-MS/MS	Danshen	Pang et al. (2018)
47	Dihydrocaffeic acid	80% methanol	HPLC-PDA	Shanzha	Sun et al. (2021a)
48	Cinnamic acid	80% methanol	HPLC-PDA	Shanzha	Sun et al. (2021b)
Triterpenoids					
49	Maslinic acid	ethyl acetate	NMR and MS	Danshen	Tung et al. (2017)
		methanol	HPLC-CAD	Shanzha	Du et al. (2023)
50	Ursolic acid	ethyl acetate	NMR and MS	Danshen	Tung et al. (2017)
		methanol	HPLC-CAD	Shanzha	Du et al. (2023)
51	Oleanolic acid	ethyl acetate	NMR and MS	Danshen	Tung et al. (2017)
		methanol	HPLC-CAD	Shanzha	Du et al. (2023)
52	Corosolic acid	methanol	HPLC-CAD	Shanzha	Du et al. (2023)
53	Betulinic acid	methanol	HPLC-CAD	Shanzha	Du et al. (2023)
54	Pomolic acid	95% ethanol	NMR	Shanzha	Zhu et al. (2022)
55	Tormentic acid	95% ethanol	NMR	Shanzha	Zhu et al. (2022)
56	Euscaphic acid	95% ethanol	NMR	Shanzha	Zhu et al. (2022)
57	Fupenzic acid	95% ethanol	NMR	Shanzha	Zhu et al. (2022)
58	α-Amyrin	95% ethanol	NMR	Shanzha	Zhu et al. (2022)
59	β-Amyrin	95% ethanol	NMR	Shanzha	Zhu et al. (2022)
60	3-Epifriedelinol	95% ethanol	NMR	Shanzha	Zhu et al. (2022)
Flavonoids					
61	Hyperoside	water	HPLC-PDA	Shanzha	Chen et al. (2013)
		50% ethanol	UPLC/Q-TOF-MS	Shanzha	Ao et al. (2020)
62	Quercetin	methanol	UPLC-Q-TOF-MS/MS	Shanzha	Ning et al. (2021)
		50% ethanol	UPLC/Q-TOF-MS	Shanzha	Ao et al. (2020)
63	Isoquercetin	water	HPLC-PDA	Shanzha	Chen et al. (2013)
		50% ethanol	UPLC/Q-TOF-MS	Shanzha	Ao et al. (2020)
64	Vitexin	methanol	UPLC-Q-TOF-MS/MS	Shanzha	Ning et al. (2021)
65	Catechin	50% ethanol	UPLC/Q-TOF-MS	Shanzha	Ao et al. (2020)
66	Epicatechin	methanol	UPLC-Q-TOF-MS/MS	Shanzha	Ning et al. (2021)
		50% ethanol	UPLC/Q-TOF-MS	Shanzha	Ao et al. (2020)
67	Hesperidin	50% ethanol	UPLC/Q-TOF-MS	Shanzha	Ao et al. (2020)
68	Rutin	methanol	UPLC-Q-TOF-MS/MS	Shanzha	Ning et al. (2021)
		water	HPLC	Shanzha	Chen et al. (2013)
		50% ethanol	UPLC/Q-TOF-MS	Shanzha	Ao et al. (2020)
69	Luteolin	50% ethanol	UPLC/Q-TOF-MS	Shanzha	Ao et al. (2020)
70	Vitexin rhamnoside	50% ethanol	UPLC/Q-TOF-MS	Shanzha	Ao et al. (2020)
71	Vitexin-2′-O-rhamnoside	methanol	UPLC-Q-TOF-MS/MS	Shanzha	Ning et al. (2021)
72	Vitexin-4′-O-glucoside	methanol	UPLC-Q-TOF-MS/MS	Shanzha	Ning et al. (2021)
73	Proanthocyanidin B2	50% ethanol	UPLC/Q-TOF-MS	Shanzha	Ao et al. (2020)
		75% ethanol	UPLC/ESI-TOF-MS	Shanzha	Qiao et al. (2014)
74	Proanthocyanidin B3	75% ethanol	UPLC/ESI-TOF-MS	Shanzha	Qiao et al. (2014)
Organic acids					
75	Citric acid	methanol	UPLC-Q-TOF-MS/MS	Shanzha	Ning et al. (2021)
		water	HPLC-PDA	Shanzha	Jiang, (2021)
76	Malic acid	water	HPLC-PDA	Shanzha	Jiang, (2021)
77	Tartaric acid	water	HPLC-PDA	Shanzha	Jiang, (2021)
78	Gallic acid	water	HPLC-PDA	Shanzha	Jiang, (2021)
79	Succinic acid	water	HPLC-PDA	Shanzha	Jiang, (2021)
80	Oxalic acid	water	HPLC-PDA	Shanzha	Jiang, (2021)
81	Linoleic acid	methanol	UPLC-Q-TOF-MS/MS	Shanzha	Ning et al. (2021)
82	Coumalic acid	methanol	UPLC-Q-TOF-MS/MS	Shanzha	Ning et al. (2021)
83	Phlogistic acid	80% methanol	HPLC-PDA	Shanzha	Sun et al. (2021a)
Volatile oil					
84	Vanillin	methanol	UPLC-Q-TOF-MS/MS	Shanzha	Ning et al. (2021)

### 4.2 Chemical constituents in Shanzha

In the last decade, approximately two hundred phytochemical compounds containing flavonoids, steroids, triterpenoids, lignans, phenylpropanoids and their glycosides, as well as other constituents such as organic acids and nitrogenous chemicals, were isolated and characterized from Shanzha (SZ), the fruit of *Crataegus pinnatifida* (Zhang et al., 2020). Among these compounds, flavonoids and organic acids, such as luteolin, vitexin, hyperoside, catechin, rutin, epicatechin and citric acid have been considered major bioactive components of SZ due to their diverse pharmacological properties. These compounds exhibited a wide range of therapeutic activities with low toxicity *in vitro* and *in vivo* (Orhan, 2018). For a comprehensive understanding of the current known structural characteristics of SZ compounds, readers are encouraged to refer to Li et al. (2023). In the present review, we just highlighted the frequent constituents (Table 1) and primary chemicals of SZ which implicated in its atherosclerotic protection (Figure 1).

### 4.3 Chemical constituents in DSF

**FIGURE 2:**
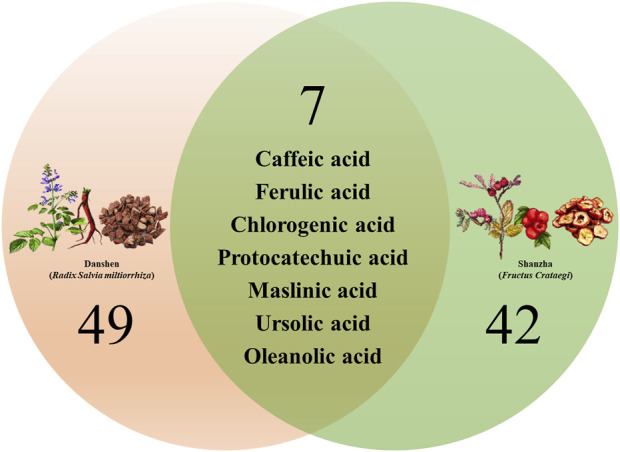
Venn diagram summary of the main chemical constituents identified from Danshen-Shanzha Formula (DSF).

We have synthesized the literature to compile a total of 84 major chemical constituents of Danshen-Shanzha Formula (DSF), as presented in Table 1 and Figure 2. Among these, there are seven components that are shared by both herbs, namely, protocatechuic acid, chlorogenic acid, caffeic acid, ferulic acid, maslinic acid, ursolic acid, and oleanolic acid. However, in most cases, the synergistic effect of a pair of herbs is unlikely to be achieved by simply mixing the chemicals from the two separate one. The composition, dissolution rate and content of chemical ingredients in herb pairs after co-decoction may differ from those of the individual decocted herbs (Wang et al., 2012). As of now, information on the overall chemical profiles of DSF remain limited. Focusing on constituents of the compatibility of each single-herb, some of the work have been conducted on the bioactive materials of DSF currently. Research has revealed that proanthocyanidin B2, salvianolic acid B, and tanshinone IIA are the active ingredients responsible for the anti-atherosclerotic effect in DSF (Pang et al., 2018). By using the HPLC tandem DAD technique, 5 components of DS (salvianolic acid A, salvianolic acid B, lithospermic acid, rosmarinic acid and danshensu) and 5 components of SZ (isoquercetin, epicatechin, proanthocyanidin B2, hyperoside and 3-caffeoylquinic acid) were obtained and identified by a fingerprint method in water decoction of DSF (Zhang et al., 2013b). On the other hand, 3 types of tanshinones (tanshinone I, tanshinone IIA and cryptotanshinone) and 2 types of triterpenic acid (oleanolic acid and ursolic acid) were detected and identified in ethanol-soluble extractives of DSF. Meanwhile, 21 different kinds of structure in DSF decoction (N-hexacosane, N-hexadecanoic acid, betulin, betulinic acid, uvaol, β-sitosterol, 4,5,4′,5′-tetrahydroxy-1,2-diphenyl ether, benzenepropanoic acid and salvianolic acid B, *etc.*) were reported by utilizing the GC-MS and LC-MS technique in earlier research of the chemical constituents. During the same period, several studies have shown that the total phenolic acids and triterpenoic acids of DSF extracts could inhibit the formation of hyperlipidemia as well as interfere with the formation of atherosclerosis in rats, which have been marked as the main chemical components of DSF for anti-oxidative and hypolipidemic effects (Chen et al., 2013). In addition, with an HPLC-based analytical approach and assays to determine antioxidant capabilities, salvianolic acid B was found to exert the primary anti-oxidative effect in the phenolic compounds of the herb pair (Yilmaz et al., 2022).

Notably, study of Zhang et al. showed that the level of hydrophilic phenolic acid components (such as salvianolic acid A, salvianolic acid B, lithospermic acid and rosmarinic acid, *etc.*) were significantly higher in DSF decoction comparing with the individual decoction of DS, while contents of proanthocyanidins and epicatechin were considerably lower than the individual decoction of SZ, presumably the compatibility of DS and SZ contributed to the solubilization of phenolic acid components in DS (Zhang et al., 2013a; Zhang et al., 2013b). Such studies could benefit for elucidating the material foundation and rationality of compatibility art of DSF, especially similar comparative studies conducted *in vivo*, however only a few works have been undertaken thus far.

## 5 Pharmacokinetic properties

Some studies about the pharmacokinetics of the single use of DS or SZ have been reported (Qu et al., 2020; Sun et al., 2022). However, research focusing on the active components and their pharmacokinetics after administration of DSF remain limited. Until now, only Liu et al. (2014) reported the pharmacokinetic behavior after the oral dosing of the combination of DS and SZ extracts. They developed a liquid chromatographic method which could simultaneously determine five active components, namely, salvianolic acid B, danshensu, hyperoside, rosmarinic acid and lithospermic acid, in the plasma of rat after oral dose of DSF. Compared to the concentration-time profiles of each components following oral dosage of the single herb and DSF to rats, all five components exhibited a short T_max_ and were rapidly absorbed *in vivo* after oral administrated the single herb, whereas T_max_ of rosmarinic acid and hyperoside were found to be shorter after dosing DSF. Besides, the C_max_ of the above five components after administration of DSF was 1.6, 2.6, 2.6, 1, and 1.7 times compared with dosing of single herb, which implied the combination of DS and SZ could quicken and enhance the absorption of their ingredients. In addition, AUCs of danshensu, rosmarinic acid, lithospermic acid, salvianolic acid B and hyperoside were elevated after oral administration of DSF, which were 4.44, 4.25, 27.3, 56.5, and 5.24 mg·h/L, respectively, whereas dosing of single herbals were 3.65, 2.09, 6.49, 38.2, and 3.84 mg·h/L, respectively (Table 2). These findings revealed significant differences in pharmacokinetics over the individual herbal and the combined use of DS and SZ. A combination of DS and SZ have the potential to improve the bioavailability and prolong the elimination of their active components in rat.

**TABLE 2:** Pharmacokinetic parameters of five primary constituents of Danshen-Shanzha Formula (DSF) after oral administration of 8.4 g/kg DSF or single herb extract (6 g/kg for Danshen extract, 2.4 g/kg for Shanzha extract) in rats.

Constituents	Oral source	Contents (mg/g)	Tmax (h)	Cmax (mg/L)	AUC_0-t_ (mg·h/L)	MRT_0–t_ (h)	T_1/2_ (h)
Danshensu	DSF	1.2	0.5	1.14 ± 5.8	3.65 ± 0.42	2.98 ± 0.15	2.85 ± 1.2
	Danshen	1.2	0.75	1.87 ± 0.17	4.44 ± 0.66	2.76 ± 0.10	3.00 ± 8.8
Rosmarinic acid	DSF	2.6	0.5	1.50 ± 0.44	2.09 ± 0.39	2.50 ± 0.44	7.52 ± 4.4
	Danshen	2.6	0.33	3.88 ± 0.41	4.25 ± 0.78	1.98 ± 0.12	4.34 ± 9.0
Lithospermic acid	DSF	5.9	0.5	3.91 ± 0.41	6.49 ± 0.88	3.27 ± 0.50	3.78 ± 3.8
	Danshen	5.9	1.0	10.0 ± 0.89	27.3 ± 3.0	2.93 ± 0.22	1.60 ± 0.67
Salvianolic acid B	DSF	48.0	0.5	13.7 ± 0.88	38.2 ± 3.0	2.85 ± 0.13	1.94 ± 2.1
	Danshen	48.0	1.5	13.2 ± 1.7	56.5 ± 6.52	3.78 ± 0.19	2.50 ± 0.45
Hyperoside	DSF	3.0	0.75	1.93 ± 0.39	3.84 ± 0.61	2.68 ± 0.16	7.0 ± 0.67
	Shanzha	3.0	0.5	3.27 ± 0.48	5.24 ± 0.79	2.22 ± 0.20	5.35 ± 3.0

The data of the contents and pharmacokinetic parameters for the five constituents of DSF, were compiled according to Liu et al.’s study (Liu et al., 2014).

## 6 Pharmacological effects

**FIGURE 3:**
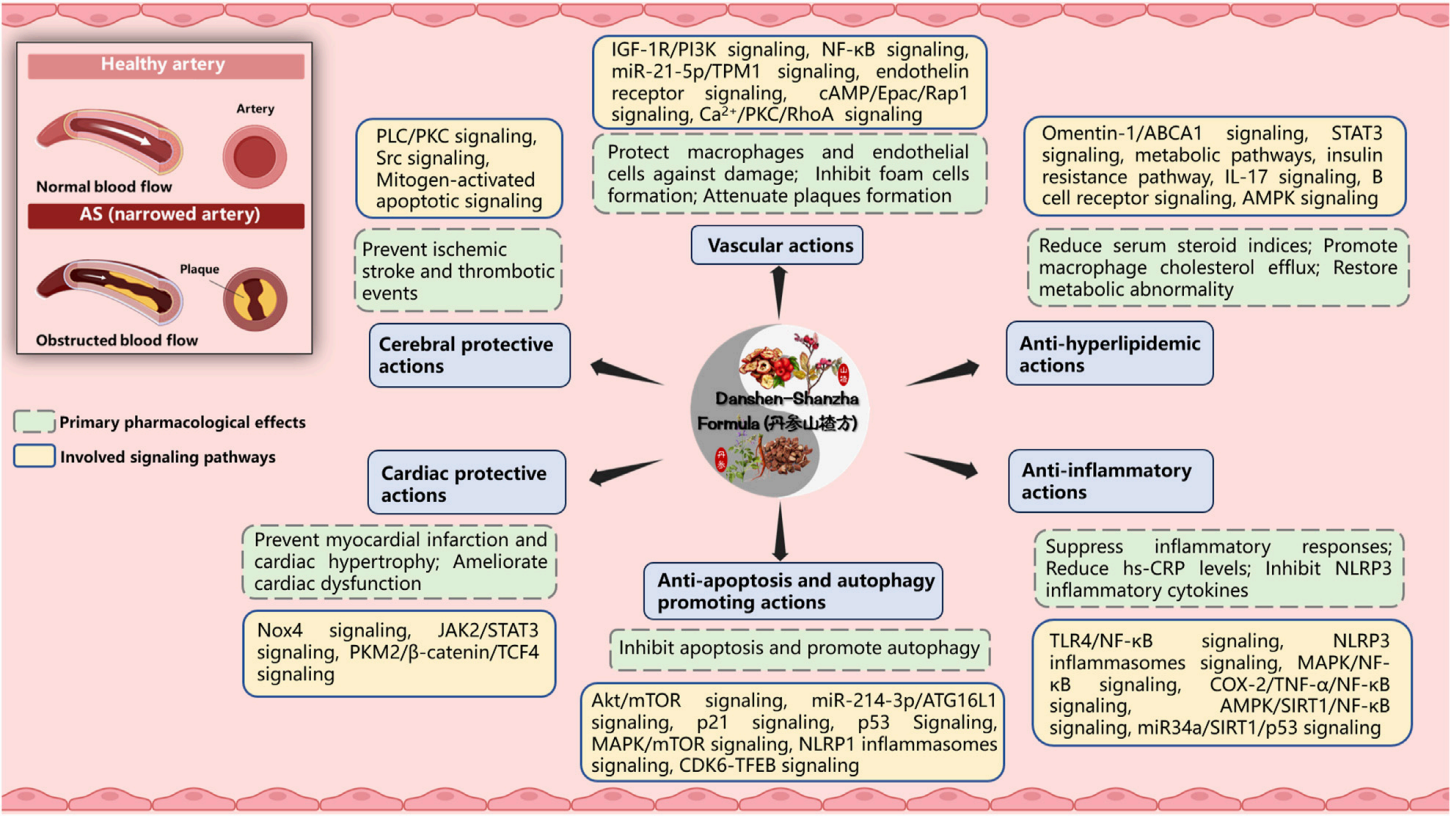
The primary pharmacological effects of Danshen-Shanzha Formula (DSF) on atherosclerosis. Image was created with BioRender.com. ABCA1, ATP-binding cassette transporter A1; AMPK, AMP-activated protein kinase; Akt, v-Akt Murine Thymoma Viral Oncogene; AS, atherosclerosis; ATG16L1, Autophagy Related Protein 16 Like Protein 1; COX-2, Cyclooxygenase-2; cAMP/Epac/Rap1, Cyclic adenosine monophosphate/exchange protein directly activated by cAMP/repressor activator protein 1; CDK6, Cyclin-dependent kinase 6; hs-CRP, hypersensitive C-reactive protein; IGF-1R/PI3K, insulin-like growth factor 1 receptor/phosphatidylinositol 3-kinase; IL-17, Interleukin-17; JAK2, Janus kinase 2; MAPK, Mitogen-activated protein kinase; mTOR, Mammalian Target of Rapamycin; NLRP, Nucleotide oligomerization domain-like receptor protein; NF-κB, Nuclear Factor-κB; Nox4, Nicotinamide adenine dinucleotide phosphate oxidase 4; P21, Cyclin-dependent kinase inhibitor 1; PLC/PKC, phospholipase C/protein kinase C; PKM2/β-catenin/TCF4, pyruvate kinase isoform M2/β-catenin/T cell factor 4; RhoA, Ras homolog gene family member A; SIRT1, silent information regulator sirtuin 1; STAT3, Signal transducer and activator of transcription 3; TFEB, Transcription factor EB; TLR4, Toll-like receptor 4; TNF-α, Tumour Necrosis Factor alpha; TPM1, tropomyosin 1.

Atherosclerosis (AS) is a chronic inflammatory diseases based on lipid metabolism disorders, affecting the blood arteries, and serves as the primary pathologic foundation for ischemic cardiovascular, cerebralvascular and peripheral vascular disorders, such as myocardial infarction, stroke, and arteriovenous thrombosis (Wu Q. et al., 2023). It represents a condition characterized by arterial narrowing and loss of arterial wall elasticity, resulting from the excessive accumulation of viscous plaque in the intima of arteries. Notably, DSF holds a prominent position as a classic representative in the realm of therapeutic theories and herbal formulas for the aforementioned disease condition within TCM. For the past few years, DSF-based preparations have been widely used for the treatment of atherosclerosis and have been important in terms of both pharmacological effects and clinical effects due to associated vascular lesions (Zhang, J. et al., 2019). Therefore, the pharmacological actions of DSF and/or its active components have been further reviewed and discussed for the aspects as follows (Figure 3 and Table 3).

**TABLE 3:** Studies regarding the pharmacological effects of the active constituents or extract from Danshen-Shanzha Formula (DSF).

Constituent/Extract	Source	Model	Effects/mechanisms	References
Vascular actions				
Tanshinone I	Danshen	Human vascular smooth muscle cells (Ang II stimulated)	Inhibit VSMC proliferation without inducing apoptosis; prevent IGF-1R/PI3K signaling activation	Wu et al. (2019a)
Dihydrotanshinone I	Danshen	ApoE^−/−^ mice (high-cholesterol/high-fat diet fed); RAW264.7 macrophages (LPS/ZVAD stimulated)	Inhibit macrophages necroptosis; enhance plaque stability	Zhao et al. (2021)
Tanshinone IIA	Danshen	Human vascular smooth muscle cells (high-glucose stimulated)	Inhibit VSMC proliferation and migration; regulate miR-21-5p/TPM1 signaling	Jia et al. (2019a)
Cryptotanshinone	Danshen	ApoE^−/−^ mice (high-cholesterol diet fed); Human umbilical vein endothelial cells (oxLDL stimulated)	Attenuate plaque formation; enhance plaque stability; inhibit NADPH oxidase subunit 4-mediated ROS generation and activation of NF-κB	Liu et al. (2015)
Danshensu	Danshen	Rats (methionine-rich diet fed)	Reduce serum homocysteine; inhibited TNF-α and ICAM-1 expression in arterial endothelia; suppress alterations of serum endothelin and NO levels	Yang et al. (2010)
Shanzha extract (WS^®^1442)	Danshen	Human umbilical vein endothelial cells (thrombin stimulated)	Inhibit detrimental effects of thrombin on adherens junctions, the F-actin cytoskeleton, and the contractile apparatus; block the calcium/PKC/RhoA signaling and activate the cAMP/Epac/Rap1 signaling	Bubik et al. (2012)
Shanzha extract	Danshen	ApoE^−/−^ mice (high-fat diet fed)	Stabilize unstable plaques; regulate inflammatory and apoptotic signaling	Wang et al. (2019)
Danshen and Shanzha extract (SC_121_)	DSF	Human umbilical vein endothelial cells (oxLDL stimulated); RAW264.7 macrophages (oxLDL stimulated)	Alleviate macrophages and endothelial cells damage; inhibit foam cell formation; reduce ROS level	Zhang et al. (2016)
Danshen and Shanzha extract	DSF	Rats (high-fat diet fed)	Increase serum nitric oxide and 6-keto-prostaglandin F1α level; decrease serum endothelin and thromboxane B2 level	Zhang et al. (2019)
Anti-hyperlipidemic actions				
Danshen and Shanzha extract	DSF	Rats (vitamin D3 stimulated plus high-fat diet fed)	Decrease serum steroid indices; elevate high-density lipoprotein cholesterols level	Zhang et al., 2013b; Zhang, 2013
Shanzha extract	Shanzha	Rats (vitamin D3 and ovalbumin stimulated plus high-fat diet fed)	Improve lipid metabolism; alleviate inflammatory cytokine responses	Zhang et al. (2013a)
Tanshinone IIA	Danshen	ApoE^−/−^ mice (high-fat diet fed); THP-1 cells and mouse peritoneal macrophages (apoA-I stimulated)	Promote cholesterol efflux; meliorates lipid accumulation; increase reverse cholesterol transport; regulate omentin-1/ABCA1 signaling	Tan et al. (2019)
Cryptotanshinone	Danshen	3T3-L1 murine pre-adipocytes	Reduce lipid accumulation; inhibit the phosphorylation of STAT3	Rahman et al. (2016)
Salvianolic acid B	Danshen	Rats (high-fat diet fed)	Regulates the expression of mRNA, circRNA and lncRNA which involved in the insulin resistance pathway, IL-17 signaling and B cell receptor signaling	An et al. (2019)
		*db/db* Mice	Decrease serum triglyceride and free fatty acid levels; regulate AMPK signaling	Huang et al., 2016
Salvianolic acid A	Danshen	Rats (high-fat diet fed); C3H10T1/2 cells	Attenuate weight gain and lipid accumulation; regulate AMPK signaling	Lai et al. (2021)
Flavonoids	Shanzha	Mice (PM_2.5_ exposure)	Increase fatty acid uptake; decrease lipid export; balance the hepatic triacylglycerol levels	Gu et al. (2023)
Shanzha extract	Shanzha	Rats (high-fat diet fed)	Restore the metabolic abnormality; regulate bio-oxidation along with metabolism of energy, amino acid and lipid pathways	Zeng et al. (2021a)
Anti-inflammatory actions				
Danshen extract	Danshen	ApoE^−/−^ mice	Decrease serum lipid levels; inhibit inflammatory responses via TLR4/NF-κB signaling	Wu et al. (2023b)
Salvianolic acid A	Danshen	Zucker diabetic fatty rats (vitamin D3 stimulated plus high-fat diet fed)	Decrease hemoglobin A1C level; ameliorate serum disrupted lipid profiles; decrease serum hypersensitive C-reactive protein level; inhibit NLRP3 inflammatory and NF-κB signaling	Ma et al. (2020)
Salvianolic acid B	Danshen	LDLR^−/−^ mice; RAW264.7 cells (LPS stimulated/oxLDL stimulated)	Decrease serum lipids levels; attenuate inflammatory cytokines; attenuate phosphorylation of MAPK/NF-κB singalongs	Zhang et al. (2022)
Tanshinones	Danshen	THP-1 macrophages (LPS stimulated	Inhibit the expression of TNF-α, IL-1β, and IL-8	Ma et al. (2016)
Cryptotanshinone	Danshen	Mice (LPS stimulated); bone marrow-derived macrophages (LPS stimulated)	Inhibit NLRP3 inflammasome activation; blocks Ca^2+^ signaling; attenuate caspase-1 activation and IL-1β secretion	Liu et al. (2021b)
Tanshinone IIA	Danshen	ApoE−/− mice (high-fat diet fed); Mouse B6 macrophages (oxLDL stimulated)	Attenuate NLRP3 inflammasome activation; downregulate IL-1β and NLRP3 expression; decrease LOX-1 and CD36 expression; inhibite NF-κB activation	Wen et al. (2021b)
		ApoE^−/−^ mice (porphyromonas gingivalis infected)	Inhibit inflammatory mediators’ secretion; downregulate miR-146b and miR-155 expressions	Xuan et al. (2017)
		ApoE^−/−^ mice (high-fat diet fed); human umbilical vein endothelial cells (oxLDL stimulated)	Attenuate buildup of plaque and the accumulation of lipids; reduce vascular inflammatory factors levels; regulate COX-2/TNF-α/NF-κB signaling	Ma et al. (2023)
Shanzha extract	Shanzha	ARPE-19 cells (high glucose stimulated)	Alleviate inflammatory, oxidative and apoptotic damages; regulate AMPK/SIRT1/NF-κB signaling and miR34a/SIRT1/p53 signaling	Liu et al. (2021c)
Quercetins	Shanzha	ApoE^−/−^ mice (high-fat diet fed); RAW264.7 cells (oxLDL stimulated)	Alleviate atherosclerotic lesions and reduce lipid retention; alleviate cellular steatosis and IL-1β secretion; suppress NLRP3 inflammasome activation; modulate galectin-3-NLR family	Li et al. (2021)
Hyperoside	Shanzha	MOVAS-1 cells (TNF-α stimulated)	Inhibit VCAM-1 expression; suppresses monocyte adhesion; suppress activation of p38 MAPK, ERK1/2, JNK, and NF-κB	Jang et al. (2018)
Anti-apoptosis and autophagy promoting actions				
Danshen extract	Danshen	ApoE^−/−^ mice (high-fat diet fed); human umbilical vein endothelial cells (oxLDL stimulated)	Attenuate formation of atherosclerotic plaque; inhibited foam cell formation and increase autophagic activity; inhibit cell proliferation and induce autophagy flux	Ko et al. (2020)
Salvianolic acid B	Danshen	RAW264.7 macrophages (cholesterol crystals stimulated)	Reduce apoptosis and proinflammatory cytokines levels; improves autophagic dysfunction; inhibit the Akt/mTOR signaling	Sun et al. (2021a)
Tanshinone IIA	Danshen	ApoE^−/−^ mice (high-fat diet fed); RAW264.7 cells (oxLDL stimulated)	Attenuate lipid accumulation and promote autophagy; regulate miR-214-3p/ATG16L1 axis; facilitate MAPK/mTOR signal-mediated autophagy	Qian et al. (2023)
Quercetin	Shanzha	RAW264.7 cells (oxLDL stimulated)	Inhibit the formation of foam cells; delay senescence; regulate mammalian sterile 20-like kinase 1 mediated autophagy	Cao et al. (2019a)
		ApoE^−/−^ mice	Enhance autophagy; upregulate P21 and P53 expression	Cao et al. (2019b)
Hypericin	Shanzha	Mice (left anterior descending ligated)	Activate autophagy; inhibit NLRP1 inflammatory pathway	Yang et al. (2021b)
		Mice (high-fat diet fed)	Increase glucose and lipid metabolism; induce lipophagy; facilitate degradation of lipid droplets; regulate CDK6-TFEB signaling	Cheng et al., 2023
Cardiac protective actions				
Tanshinone IIA	Danshen	Rats (left anterior descending ligated)	Reduce the expression of collagen families, ameliorate myocardial fibrosis and cardiac dysfunction; regulate Nox4 signaling	Chen et al. (2021)
		Mice (left anterior descending ligated)	Reduce the release of inflammatory cytokines; inhibit cell apoptosis; promote angiogenesis	Wu et al. (2019b)
Protocatechuic aldehyde	Danshen	Rats (isoproterenol stimulated); neonatal rat cardiomyocytes (isoproterenol stimulated)	Downregulate hypertrophic gene markers; reduce cardiomyocyte cross-sectional area and collagen level; inhibit the JAK2/STAT3 signaling	Fang et al. (2018)
		Rats (isoproterenol stimulated); neonatal rat cardiomyocytes (oxygen/glucose deprived; hydrogen peroxide stimulated)	Reduce lipid peroxidation and DNA damage; prevented cell apoptosis; protect cell survival; regulate PKM2/β-catenin/TCF4 signaling	Wu et al. (2021)
Salvianolate	Danshen	Rats (left anterior descending ligated); neonatal rat cardiomyocytes (Ang II stimulated)	Improve cardiomyocyte remodeling; downregulate the expression of β-MHC, reduce nuclear NFATc3 translocation; downregulation of CaNA subunit expression; inhibit CaN activity	Chen et al. (2022)
Shanzha extract	Shanzha	Rats (isoproterenol stimulated)	Decrease myocardial enzyme indexes level; Decrease blood liquid indexes level	Ao et al. (2020)
Cerebral Protective Actions				
Danshen and Shanzha extract	DSF	Rats (Rose Bengal injected and cold-light source irradiated)	Regulate vascular endothelial functions and inflammatory factors; regulate protein expression of vWF, VCAM-1, and ICAM-1; downregulate gene expression of ICAM-1	Ding et al. (2020)
Danshen extract	Danshen	Rats (middle carotid artery occluded)	Inhibit thrombosis formation and platelet aggregation; activate PLC/PKC signaling	Fei et al. (2017)
Salvianolic acid A	Danshen	Rats (middle cerebral artery occluded); human brain microvascular endothelial cells (oxygen/glucose deprived)	Improve neurological deficits, intracerebral hemorrhage, BBB disruption, and vascular endothelial dysfunction; suppress degradation of tight junction proteins; blocked the activation of the Src signaling	Liu et al. (2021a)
Vitexin	Shanzha	Mice (common carotid artery ligated); primary cortical neuronal cells (oxygen/glucose deprived)	Alleviate neurological impairment; decrease cerebral infarct volume; mitigate neuronal damage, inhibit phosphorylation of Ca^2+^/Calmodulin-dependent protein kinase II; decrease protein expressions of NF-κB and cleaved caspase-3 levels	Min et al. (2017)

Abbreviations: Ang II, Angiotensin II; ApoE, Apolipoprotein E; ABCA1, ATP-binding cassette transporter A1; AMPK, AMP-activated protein kinase; Akt, v-Akt Murine Thymoma Viral Oncogene; BBB, Blood-brain barrier; COX-2, Cyclooxygenase-2; cAMP/Epac/Rap1, Cyclic adenosine monophosphate/exchange protein directly activated by cAMP/repressor activator protein 1; CDK6, Cyclin-dependent kinase 6; ERK, Extracellular signal-regulated kinase; IGF-1R/PI3K, insulin-like growth factor 1 receptor/phosphatidylinositol 3-kinase; IL-17, Interleukin-17; JNK, c-Jun N-terminal kinase; JAK2, Janus kinase 2; LPS, lipopolysaccharide; MAPK, Mitogen-activated protein kinase; MOVAS-1, Mouse vascular smooth muscle cell line 1; mTOR, mammalian target of rapamycin; NLRP3, Nucleotide oligomerization domain-like receptor protein 3; NF-κB, Nuclear Factor-κB; NFATc3, Nuclear factor of activated T cells cytoplasmic 3; Nox4, Nicotinamide adenine dinucleotide phosphate oxidase 4; oxLDL, Oxidized low-density lipoprotein; PM_2.5_, Particulate matter 2.5; P21, Cyclin-dependent kinase inhibitor 1; PLC/PKC, phospholipase C/protein kinase C; PKM2/β-catenin/TCF4, pyruvate kinase isoform M2/β-catenin/T cell factor 4; RhoA, Ras homolog gene family member A; ROS, reactive oxygen species; STAT3, Signal transducer and activator of transcription 3; TFEB, Transcription factor EB; THP-1, human leukemic monocyte; TLR4, Toll-like receptor 4; TNF-α, tumour necrosis factor alpha; TPM1, tropomyosin 1; VSMC, vascular smooth muscle cell; VCAM-1, Vascular cell adhesion molecule-1; vWF, Von Willebrand factor.

### 6.1 Vascular actions

The primary pathological changes involve impairment of vascular endothelial cells and excessive lipid accumulation in the walls of blood vessels. It has been proven that various disruptions in vascular homeostasis occur during the onset of atherosclerosis, such as abnormal proliferation or death of endothelial and smooth muscle cells, whereas several studies have revealed the beneficial effects of both quinones and phenylpropanoids from DSF in preserving vascular homeostasis. Tanshinone I, a lipophilic o-phenanthrenequinone compound from DS, was found to inhibit cell proliferation, migration, tube formation, and vessel sprouting in basal and Ang II-stimulated vascular smooth muscle cells (Wu et al., 2019). Dihydrotanshinone I, one of another active ingredients of DS, was demonstrated to stabilize vulnerable AS plaques through the suppression for RIP3-mediated necroptosis of macrophages (Zhao et al., 2021). Tanshinone IIA has the potential to substantially inhibit the proliferation as well as the migration of vascular smooth muscle cells (VSMCs) (Jia S. et al., 2019). Cryptotanshinone has been found to exhibit considerable anti-atherosclerotic activity, as it significantly attenuates atherosclerotic plaques formation as well as improves plaque stability in ApoE^−/−^ mice through regulating the expression of LOX-1 and MMP-9, along with the inhibition of generated ROS and activated NF-κB signalling (Liu et al., 2015). Long-term administration of danshensu has been found to have the potential to prevent or attenuate the development of atherosclerosis. This effect may be attributed to the suppression of pro-inflammatory cytokine and adhesion factors in the arterial endothelial cells, as well as alterations in homocysteine levels and circulating molecules that regulate vascular contraction and relaxation through endothelial cells, such as NO and endothelin (Yang et al., 2010). The vascular endothelial permeability is regarded as an early indicator of vascular injury and one of the pivotal factors contributing to the development of atherosclerotic disease. The dried extract of Shanzha, known as WS^®^1442, demonstrated efficacy in ameliorating endothelial hyperpermeability and preventing endothelial dysfunction by augmenting crucial factors such as adhesive junctions, actin cytoskeleton, and cell contractile apparatus in human umbilical vein endothelial cells (HUVECs), where the mechanism is potentially attributed to the inhibition of the calcium/PKC/Rho signaling responsible for barrier instability and activation of the cAMP/Epac1/Rap1 signaling related to barrier-stability (Bubik et al., 2012). Shanzha extract also exhibits both anti-atherosclerotic properties and stabilizing effects on unstable plaques. The underlying mechanisms may involve modulation of inflammatory and apoptotic signaling pathways (Wang et al., 2019). Furthermore, Zhang et al. (2016) observed a significant alleviation of ox-LDL-induced damage in HUVEC and RAW264.7 cells upon administration of SC_121_, a kind extract of DSF, demonstrating a dose-dependent reduction in reactive oxygen species levels and foam cell formation. These findings suggest that DSF possesses the capability to inhibit endothelial cell damage and attenuate oxidative stress. DSF was also demonstrated to be able to protect endothelial functions by regulate multiple signaling, including estrogen signaling system, ErbB signaling, VEGF signaling, along with FoxO signaling. By using DSF aqueous decoction, NO and 6-keto-prostaglandin F1α levels were elevated in rat serum, whereas endothelin along with TXB2 levels were found to be lowered, suggesting that endothelium protection could be one of the role by which DSF plays in its anti-atherosclerotic mechanisms (Zhang et al., 2019). Besides, several preparations containing DSF as the core of their foundation have also demonstrated notable vaso-protective properties, such as Baoyuan Huoluo Prescription (Xu et al., 2022) and Yirui capsule (Dai et al., 2016). The inclusion of DSF as foundational herbs in these preparations underscores the growing research emphasis on harnessing the therapeutic properties of this formula for the preservation of vascular health. However, with regards to these DSF-containing preparations, it is now imperative to primarily focus on elucidating the precise mechanism underlying their vascular protective effects and assessing their long-term efficacy.

### 6.2 Anti-hyperlipidemic actions

Dyslipidemia is another indicator of risk for atherosclerotic and associated cardiovascular disorders, whereas several studies have shown the benefits of DS and SZ extracts for the regulation of lipid level in human body (Liu et al., 2022b; Li, 2023). DSF was demonstrated to alleviate atherosclerotic symptoms in dyslipidemia rat induced by vitamin D_3_ stimulation plus high-fat-diet-fed, as it could lower serum steroid indices including total cholesterols, triglycerides as well as low-density lipoprotein cholesterols, whereas elevating high-density lipoprotein cholesterols level (Zhang et al., 2013a; Zhang et al., 2013b; Zhang, 2013). Tanshinone IIA, one of the primary constitutes of DS, has been demonstrated to promote macrophage cholesterol efflux through the omentin-1/ABCA1 signaling, thereby preventing atherosclerotic conditions in apoE^−/−^ mice (Tan et al., 2019). Cryptotanshinone, another important lipophilic ingredient of DS, exerted anti-adipogenic effects through regulating STAT3 in the early stages of adipogenesis (Rahman et al., 2016). Salvianolic acid B, the major hydrophilic constituent of DS, exhibits anti-hyperlipidemic effect by reducing blood triglyceride and free fatty acid levels, while also modulating the expression profiles of mRNA, circRNA, and lncRNA in high-fat diet-induced obesity and dyslipidemia, respectively (Huang et al., 2016; An et al., 2019). Similarly, Sal A enhances browning of white adipose tissue in male mice fed with high-fat diet and in cultured adipocytes, suggesting its potential as another hydrophilic component of DS for the treatment and/or prevention of obesity (Lai et al., 2021). Additionally, the flavonoids of SZ have attracted great research interests due to their potential to treat dyslipidemia, obesity and atherosclerosis by inhibiting the enzyme acyl-coA cholesterol acyltransferase, thereby reducing the level of very low density lipoprotein and low density lipoprotein cholesterol to reduce atherosclerotic lesion areas (Dehghani et al., 2019; Gu et al., 2023). Zeng et al. (2021) found that n-butanol and ethyl acetate extracts of SZ, with the primary ingredients such as chlorogenic acid, hyperoside, isoquercitrin, rutin, vitexin and quercetin, exhibited excellent efficacy on hyperlipidemia rats, influenced the metabolic alterations of adipose tissue, and restored the metabolic abnormality in plasma via modifying bio-oxidation, along with energy, amino acid, as well as lipid metabolism pathways. Collectively, accumulating evidence supports the notion that DSF possesses the potential to ameliorate hyperlipidemia. However, due to variations in dosage, animal models, treatment duration, and administration routes, it remains challenging to evaluate the constituents involved in lipid level regulation. Furthermore, despite significant progress in *in vivo* studies, further investigation into the anti-hyperlipidemic effects of DSF and its active components, as well as elucidation of underlying mechanisms, is necessary using experimental models that more closely resemble humans and clinical settings.

### 6.3 Anti-inflammatory actions

Atherosclerosis-associated inflammations is mediated through proinflammatory cytokines, inflammatory signaling, as well as bioactive lipids and adhesion molecules (Zhu et al., 2018). Active ingredients of DSF have been reported to act as regulators on the expressions of several genes involved in anti-inflammation processes. *Salvia miltiorrhiza* aqueous extract was demonstrated to exert the preventive effect to the occurrence of early atherosclerosis by reducing blood lipids as well as suppressing inflammatory responses through TLR4/NF-κB signalings (Wu et al., 2023). The preventive benefits of Sal A against early atherosclerosis have also been demonstrated by Ma *et al.* They conducted a study to investigate the role of Sal A in male ZDF rats with metabolic dysfunctions induced by a high-fat diet and vitamin D3 injection. The study found that supplementing with Sal A significantly reduced disruptions, leading to a substantial decrease in blood cholesterol, low-density lipoprotein, and triglyceride levels. These findings suggest that Sal A may play a crucial role in maintaining cardiovascular health by reducing risk factors associated with early atherosclerosis. Another notable finding of this study was the observed decrease in serum hs-CRP levels with Sal A administration. Elevated hs-CRP levels indicate inflammation and are biomarkers for chronic diseases, including cardiovascular disease and diabetes-related complications. The ability of Sal A to reduce hs-CRP suggests its potential anti-inflammatory properties, which may contribute to improved overall health outcomes. The protective action exhibited by Sal A against metabolic dysfunctions is believed to be attributed to its inhibition of NLRP3 inflammatory cytokines activation along with NF-κB signal pathways modulation (Ma et al., 2020). Salvianolic acid B, as another primary hydrophilic components of DS, has been reported to reduce inflammatory cytokine levels as well as attenuate the phosphorylation of MAPK/NF-κB signalings in RAW264.7 cells triggered by oxidized low-density lipoprotein/lipopolysaccharide, and thereby exhibit anti-inflammatory consequences against atherosclerosis *in vitro* and *in vivo* (Zhang et al., 2022). Meanwhile, it has been demonstrated that the lipophilic components of DS (tanshinones) possess anti-inflammatory activities that considerably reduce levels of TNF-α, IL-1β as well as IL-8 in tohoku hospital pediatrics-1 (THP-1) macrophages stimulated by lipopolysaccharide (Ma et al., 2016). According to Liu et al. (2021) study, cryptotanshinone was proven to be a selective agonist of NLRP3 inflammasome, thus inhibiting the activation of caspase-1 as well as the formation of IL-1β in animal model of NLRP3 inflammasome-mediated disorders. Similarly, tanshinone IIA has also been shown to suppress the activated NLRP3 inflammasomes in high-fat diet fed ApoE^−/−^ mice (Wen et al., 2021), and inhibit the secretion of inflammatory mediators like C-reactive protein, TNF-α, oxidized low-density lipoprotein as well as IL-1β in serum of apolipoprotein E knockout mice (Xuan et al., 2017), thereby alleviating processes of atherosclerosis. A recent study also demonstrated that tanshinone ⅡA could attenuate the buildup of plaque and the accumulation of lipids as well as reduced vascular inflammatory cytokines levels in both ApoE knocked out mice and oxidized low-density lipoprotein-stimulated HUVECs, while these protective effects may partly associate with the regulation of COX-2/TNF-α/NF-κB signal pathways (Ma et al., 2023). On the other hand, the polyphenol extracts of SZ alleviated high glucose-induced inflammatory, oxidative as well as apoptotic damages in ARPE-19 cells through the regulation of AMPK/SIRT1/NF-κB pathway along with the suppression of miR-34a/SIRT1/p53 signaling axis (Liu et al., 2021). In addition, Li et al. (2021) have found that inflammatory lesion of the high-fat diet treated ApoE^−/−^ mouse are triggered by Gal-3 activation of the NLRP3 inflammasomes, which serves as a potential site for quercetins that exerts advantageous anti-atherogenic actions, whereas indicating an potential path for preventing and treating atherosclerosis by natural-derived quercetins. Also, hyperoside, a quercetin derived glycosides of SZ, has been shown to inhibit TNF α-mediated vascular inflammation in MOVAS-1 cell lines by the downregulation of MAPKs-NF-κB signal axis (Jang et al., 2018). In addition to the aforementioned signaling pathways, further investigation is warranted to explore the potential of DSF in modulating other crucial anti-inflammatory mechanisms associated with atherosclerosis. For instance, studies could focus on the impact of DSF on monocyte/macrophage polarization towards an anti-inflammatory M2 phenotype and its influence on T-cell differentiation or regulatory T cell function during atherogenesis. Moreover, the identification of specific active components within DSF that are related to the observed anti-inflammatory activities could enhance our understanding of its therapeutic potential against inflammation associated with atherosclerosis. Isolating and characterizing these compounds could also pave the way for developing targeted therapies or novel drug candidates.

### 6.4 Anti-apoptosis and autophagy promoting actions

Progressive apoptosis are the major events that occur during processes of atherosclerosis (Sun et al., 2021). Vascular endothelial cells that undergo excessive apoptosis will lose their integrity and thus triggers the release of several cell adhesion molecules, resulting in the promotion for the conversion of monocytes into macrophages and further into foam cells, which in turn stimulates the proliferation of smooth muscle cells and the ultimate development of atherosclerotic plaques. On the other hand, it is well accepted that mild activated autophagy could minimize oxidative injure, inflammatory damage, and lipid buildup, as well as prolong the time for the formation of plaques during atherosclerotic plaque development. Hence, regulating apoptosis and autophagy could be an effective approach for preventing or delaying atherosclerosis (Zhou et al., 2023). DS extracts have been observed to promote autophagy as well as prevent senescence in both human umbilical vein and aortic endothelial cell lines, where it could activate the AMPK signaling and its anti-aging benefits are abolished when AMPKα is suppressed. Also, it has been shown that DS extracts exhibit the activities to trigger autophagy which were exert by LC3 transformations and p62 degradations, decrease the release of pro-inflammatory cytokines in the blood and thus mitigated the degeneration of the aorta in atherosclerosis mouse models, all the actions helped to lower the incidence of atherosclerotic plaques (Ko et al., 2020; Liu et al., 2023). In order to investigate the potential association between Sal B and autophagy as well as apoptosis, Sun et al. established a macrophage model of atherosclerosis induced by cholesterol crystals. They subsequently demonstrated that treatment with Sal B significantly augmented autophagy impairments in macrophages, attenuated the rate of macrophage apoptosis, and inhibited the accumulation of proinflammatory factors secreted by injured macrophages. These effects were mediated through regulation of the Akt/mTOR signaling pathway (Sun et al., 2021). In fact, both phenylpropionic acids and their polymers in DSF possess potential as regulators of apoptosis and/or autophagy, potentially attributed to the presence of polyhydroxyl groups within their molecular structures. Furthermore, a recent study has demonstrated that Tanshinone IIA effectively mitigates atherosclerosis by attenuating lipid accumulation and promoting autophagy in ApoE^−/−^ mice fed with a high-fat diet. The underlying mechanisms may involve the regulation of the miR-214-3p/ATG16L1 axis, thereby facilitating MAPK/mTOR signal-mediated autophagy to alleviate AS (Qian et al., 2023). In contrast to the promotion of autophagy, the inhibitory effect on excessive activation of autophagy was also observed in rats treated with salvianolate 3 days after ischemia/reperfusion (Yang et al., 2021). Besides, similar pharmacological activities were also demonstrated in SZ, another composed herb of the DSF formula, with the results of a recent systemic pharmacology study suggesting that the flavonoids and their glycosides in SZ may have potential regulatory effects on oxidative stress-induced apoptosis and autophagy, and these effects are mediated through multiple targets and pathways (Huang et al., 2023). In corroboration, previous research has confirmed the efficacy of quercetin in inhibiting the production of foam cells triggered by oxidized low-density lipoprotein and delaying senescence. It is speculated that the mechanism of action could be associated with the regulatory effect for mammalian sterile 20-like kinase 1 mediated autophagy in Raw264.7 cells (Cao et al., 2019a). Furthermore, the attenuation on atherogenesis by quercetin that linked with the enhancement of autophagy have also been proved in ApoE^−/−^ mice, as seen by the activated expression of P21 as well as P53 (Cao et al., 2019b). Hypericin, also a flavonoid glycolide derived from SZ, has been shown to play a cardioprotective role by activating autophagy and inhibiting the NLRP1 inflammatory pathway, which improves myocardial hypertrophy and fibrinogen deposition after myocardial infarction in mice (Yang et al., 2021). Additionally, hypericin induces lipophagy, a specific form of autophagy that facilitates the degradation of lipid droplets, and partial inhibition of autophagy results in reduced expression of uncoupling protein 1 (Cheng et al., 2023). Ultimately, the significant clinical implications of these findings warrant further investigation. If subsequent preclinical trials validate the safety and efficacy profile of DSF-containing preparations or compounds derived from DSF as observed in the aforementioned study, they have the potential to emerge as an innovative pharmaceutical for atherosclerosis treatment. The anti-apoptotic properties of DSF could prevent cell death within arterial walls, while its ability to promote autophagy may assist in clearing lipid deposits responsible for plaque formation. However, it is important to note that additional research is required to fully elucidate the mechanisms underlying DSF’s effects on apoptosis and autophagy regulation.

### 6.5 Cardiac protective actions

Acute coronary artery occlusion is often associated with atherosclerosis and plaque rupture, leading to myocardial ischemia or even infarction (Mehta et al., 2022). An enormous number of laboratory and clinic studies have shown that a series of formulas based on the DSF can improve cardiac function and morphology after acute myocardial infarction, indicating a promising myocardial protective effect of DSF (Lv et al., 2022; Sun and Zhang, 2022; Xu et al., 2022). Several studies have demonstrated that DS (either as a prodrug or as the formulation) has a beneficial effect on the heart during the pathological process of atherosclerosis both at progression stage and acute exacerbation stage (Liu et al., 2022c). It has been established that tanshinone IIA considerably ameliorate the myocardial fibrosis and cardiac dysfunction brought on by post-infarction heart failure in rats. As observed in the experiment that significant reductions in the expression of collagen families, MMP families, TGF-β and α-SMA both in the hearts of infarcted rats and Ang II-triggered cardiac fibroblasts cultured *in vitro* after tanshinone IIA intervention, whereas the mechanism may partly attributed to the modulation of nox4 signalling (Chen et al., 2021). Tanshinone IIA has also been implicated in myocardial protection through anti-inflammatory, anti-apoptotic, and anti-angiogenic effects in some other studies (Wu et al., 2019). Protocatechuic aldehyde, one of the most important hydrosoluble phenylpropanoic acid ingredients of DS, prevents ISO-induced myocardial infarction and cardiac hypertrophy by modulating β-catenin/TCF4 and JAK2/STAT3 signal cascades, respectively, which implies that it plays an important role in the cardioprotective actions of DS and its related fomulations (Fang et al., 2018; Wu et al., 2021). The protective effects of salvianolate on cardiomyocyte remodeling after myocardial infarction have also been reported, with the mechanism involving regulation of the calcineurin/nuclear factor C3 pathway in activated T cells and B-myosin heavy chain (Chen et al., 2022). Quercetin, as an important flavonoid component in SZ, has always received attention in the research area of cardiovascular disease prevention. In recent years, quercetin has been demonstrated to have cardioprotective properties via a variety of approaches, including anti-oxidative stress, anti-inflammation, and anti-apoptosis. Furthermore, quercetin is capable of regulating myocardial electrophysiological processes such as endothelin-1 receptor inhibition, NO stimulation improvement, large conductance calcium-activated potassium channels activation, and antagonism of Ca^2+^ overload, *etc.*, to induce vasodilation effects and thus exerting an myocardial protective role (Zhang et al., 2020). In addition, study of Ao et al. indicated that phenylpropionic acids, triterpenic acids, tannins, along with flavonoids might be the chemical underpinnings of SZ for the protection of ischemic heart (Ao et al., 2020).

### 6.6 Cerebral protective actions

Ischemic stroke is a kind of acute cerebrovascular disorder resulting from blood vessel rupture or occlusion. To reduce the risk of stroke, it is important to consider the state of blood coagulation as well as vascular protection (Carlin et al., 2022). The protective effect of DSF on cerebrum has been clearly demonstrated in a rat model of cerebral infarction. As evidenced by the elevated levels of blood perfusion, serum tissue plasminogen activator as well as 6-keto-PGF1α, and the reduced protein expression levels of vascular cell adhesion factors such as ICAM-1 along with VCAM-1, in focal cerebral infracted rats treated with DSF plus with swimming exercise. The above findings of Ding et al. indicate that DSF treatment combined with swimming may protect against focal cerebral infarction by regulating the interactions involving endothelial function and inflammatory cytokines, whereas it further implies a promising role and potential application for the use of DSF-related preparations combined with exercise in the prevention and treatment of thrombosis (Ding et al., 2020). Both DS and SZ serve as important role in the cerebroprotective effects of DSF, as several previous studies revealed that salvia quinones and phenylpropionic acids, along with hawthorn flavonoids are all contribute to the protective actions against ischemic stroke. In a study conducted by Fei et al. (2017), it was demonstrated that tanshinones extracts had the potential to reduce the risk of thrombotic events and platelet aggregation through activating the PLC/PKC signaling axis, thereby alleviating permanent middle cerebral artery occlusion-induced cerebral ischemia in rats. According to Liu et al. (2021)’s work, increased cell viability and inhibition for the degradation of tight junction proteins were observed in human brain microvascular endothelial cells with OGD injure after treatment of salvianolic acid A. They also found that salvianolic acid A blocked the activation of the Src signaling pathway *in vivo* and *in vitro*, as well as reversed the elevated levels of matrix metalloproteinases subsequent to a cerebral ischemia, and thus speculating salvianolic acid A may be one of the potential therapeutic basis of DS for the prevention of ischemic stroke. Furthermore, vitexin pretreatment substantially alleviated neurological impairment, decreased cerebral infarct volume, and mitigated neuronal damage thus protecting the brain from cerebral I/R injury, and this effect may be mediated by mitogen-activated apoptotic signaling (Min et al., 2017).

Overall, the traditional therapeutic system of TCM, in contrast to western medicine, is founded upon centuries of clinical practice and provides a robust basis for pharmacological research of TCM formula. However, the complexity of herbal formula presents a challenge in identifying its specific chemical components that mediate activation and subsequent affected node in the pathophysiological process. Given that multiple compounds within herbal formulas may exert distinct roles in pathophysiology by activating or inhibiting various targets, some of which might even counteract therapeutic outcomes, a potential solution lies in simplifying the formula to enhance targeting and specificity for specific diseases. In recent years, researchers have been exploring a novel approach to elucidate the pharmacological mechanism of TCM as well as develop innovative formulas known as “component-based Chinese medicine” (Zhang et al., 2015). This approach involves substituting the primary formula with a combination of multiple active components in the form of a new type of TCM formula. The resulting formula exhibits typical chemical, pharmacological, and pharmacokinetic characteristics due to its well-defined chemical composition, while still adhering to the theoretical principles of TCM prescriptions (Wang et al., 2008; Qi et al., 2019).

## 7 Conclusion and prospects

As per the review presented herein, we provided a referential basis for exploring the use of *Radix Salvia miltiorrhiza* and *Fructus Crataegi* as a Chinese herb pair formula (Danshen-Shanzha Formula) for treating cardiovascular diseases associated with atherosclerosis by summarizing the comprehensive information about this formula. The Danshen-Shanzha Formula (DSF), a well-established herb pair and herbal formula in traditional Chinese medicine systems, has long held significant importance. Phenylpropanoids, polysaccharides, triterpenic acid, organic acids, flavonoids and their derivatives are proposed to be the primary bioactive components of DSF. This formula exhibits significant clinical effects on atherosclerosis, based on the TCM concept of promoting blood circulation and removing blood stasis with strengthening stomach function and eliminating turbidity, which can be exerted significant therapeutic effects even at the chronic stage of atherosclerosis with less side-effect. Therefore, DSF holds great potential to be developed as an innovative modern formulation to obtain superior remedies that have both high safety and effectiveness for the treatment of atherosclerosis.

However, in order to further develop DSF as innovative therapeutic agents for atherosclerosis and shed light on the compositive principle and action features of DSF, a number of obstacles arising from common TCM concerns have to be overcome. Firstly, the standard preparation method for DSF has to be developed, unified and followed as to achieve optimized pharmacological properties. And also, the successful development of standardized preparation of DSF holds great importance in its transformation into a commercially viable product that adheres to the international standards. In terms of developing DSF as a standardized decoction, several crucial technical challenges urgently need to be standardized and unified. These challenges include the establishment of a standardized preparation procedure and quality control measures for the decoction of DSF, the insurance for the consistency between the quality and efficacy evaluation of standardized DSF decoction, and the quantity marker value transmission of quality marker in individual herbs, decoction pieces and standard decoctions, etc (Chi et al., 2019). In addition, the combinational use of DS and SZ was deemed to be the main reason for the increased pharmacological effects of DSF. Despite TCM theory’s illustrations of the art of their compatibility, whereby DS and SZ would be attributed to activating blood circulation and reducing lipids, respectively, their compatibility mechanism remains currently seldom revealed. Thus, by employing the modern biological techniques and pharmacological approaches, studies focusing on clinic syndromes together with the subsequent development of preclinic study system *in vitro* and *in vivo* with consistent pathological features, pharmacokinetical behaviour and biomarkers are expected to elucidate the functional mechanisms as well as the rationality and rule for the compatibility art, such as potentiation and assistance in the prescriptions of herb pairs, and also to further reveal the concepts like “remove blood stasis” and “detoxifying” in TCM theories (Qi et al., 2019; Guo et al., 2023). Moreover, despite centuries of application, it is critical to perform more extensive clinical studies towards the advancement of mechanism-based on evidence-based medicine on the safety application of DSF, which will provide more comprehensive scientific and authoritative evidence regarding the effectiveness of this herbal formula (Zeng X. et al., 2021).

In conclusion, DSF is a representative of TCM herb pair for the treatment of atherosclerotic diseases, however there are some drawbacks. Future research should concentrate on the improvement of quality control, the elucidation of functional mechanisms, as along with the reconfirmation of clinical effectiveness and safety.

## Abbreviations

APN, Adiponectin; ADME, Absorption, Distribution, Metabolism and Excretion; AS, Atherosclerosis; AUC, Area Under Curve; CNKI, China National Knowledge Infrastructure; DSF, Danshen-Shanzha Formula; DS, Danshen; SZ, Shanzha; DSHP, Danshen and Shanzha herb pair; ErbB, Erythroblastic Oncogene B; ET, Endothelin; EGFR, Epidermal Growth Factor Receptor; HPLC-PDA, High Performance Liquid Chromatography with Photodiode Array detector; HPLC-UV, High Performance Liquid Chromatography with Ultraviolet detector; ICAM-1, Intercellular Cell Adhesion Molecule-1; IL, Interleukin; IMT, Intima-Media Thickness; LOX-1, ectin-like oxLDL receptor-1; NO, nitric oxide; ROS, reactive oxygen species; STAT3, signal transducer and activator of transcription 3; TCM, Traditional Chinese Medicine; TNF, Tumor Necrosis Factor; TXB2, thromboxane B2; VEGF, Vascular Endothelial Growth Factor; VCAM-1, Vascular Cell Adhesion Molecule-1; ZDF rat, zucker diabetic fatty rat.
